# Influenza A(H3N2) virus variants and patient characteristics during a summer influenza epidemic in Taiwan, 2017

**DOI:** 10.2807/1560-7917.ES.2017.22.50.17-00767

**Published:** 2017-12-14

**Authors:** Tsung-Pei Tsou, Chia-Ping Su, Wan-Ting Huang, Ji-Rong Yang, Ming-Tsan Liu

**Affiliations:** 1 Division of Preparedness and Emerging Infectious Diseases, Centers for Disease Control, Ministry of Health and Welfare, Taipei, Taiwan; 2 Office of Preventive Medicine, Centers for Disease Control, Ministry of Health and Welfare, Taipei, Taiwan; 3 Institute of Epidemiology and Preventive Medicine, College of Public Health, National Taiwan University, Taipei, Taiwan; 4 Center for Research, Diagnostics and Vaccine Development, Centers for Disease Control, Ministry of Health and Welfare, Taipei, Taiwan

**Keywords:** influenza, influenza virus, vaccine-preventable diseases, laboratory surveillance, epidemiology

## Abstract

We report a summer influenza epidemic caused by co-circulation of multiple influenza A(H3N2) variants in clade 3C.2a. Compared with other clades, a putative clade 3C.2a.3a was more commonly isolated from severely ill patients; 3C.2a.4 was more commonly isolated in outbreak cases. Time from vaccination to illness onset was significantly shorter in severely ill patients infected with clade 3C.2a.3; characteristics and outcomes of patients infected with different clades were similar. No resistance to antiviral medications was found.

Recently, influenza A(H3N2) virus variants carrying substitutions N121K, S144K and T135K have been reported in Canada, Denmark, Israel and the United Kingdom (UK), causing outbreaks during the northern hemisphere 2016/17 influenza season and suboptimal vaccine effectiveness (VE) [1-5]. Information on clinical characteristics of patients infected with these variants is lacking, and the impact on public health practice remains unknown.

In Taiwan, the influenza season usually starts from December, and peaks in January to February of the following year [6]. The 2016/17 influenza season in Taiwan has been characterised by an unusual summer peak, which started in mid-May, peaked in late June and returned to baseline in August, with predominant circulation of influenza A(H3N2) viruses [7]. We performed a phylogenetic analysis of the variants and analysed the characteristics of patients with severe illness to fill the gap between knowledge about virological characteristics and their possible implications for public health practice.

## Collection and testing of respiratory samples

The Taiwan Centers for Disease Control (TCDC) in Taipei has a national influenza laboratory surveillance network collecting respiratory specimens from three different sources to monitor changes in the circulating influenza strains. Samples from outpatients with influenza-like illness (ILI), defined as fever ≥ 38.0 °C plus respiratory symptoms and at least one of the following: myalgia, headache, general malaise, are collected by sentinel doctors (general practitioners in local clinics, or doctors in medical centers collecting specimens from their outpatients). Samples from patients involved in suspected influenza outbreaks are collected by public health workers in 22 city/county health bureaus. Severe influenza requiring intensive care is a notifiable disease in Taiwan; samples from severe cases are collected by physicians in the hospitals. All specimens obtained by the three different sources are sent to one of the eight contract laboratories or TCDC central laboratory for real-time RT-PCR and viral culture [8]. All viruses isolated by cell culture are sent to the central laboratory for genotyping, antigenic characterisation, and antiviral resistance testing. Detailed methods have been previously described [8].

### Sequencing results

From 1 July 2016 to 15 July 2017, 720 of 2,228 influenza A(H3N2) isolates were sequenced. The isolates originated from outpatients with ILI (n = 237; 32.9%), severe cases (n = 300; 41.7%) and outbreak cases (n = 183; 25.4%).

**Figure 1:**
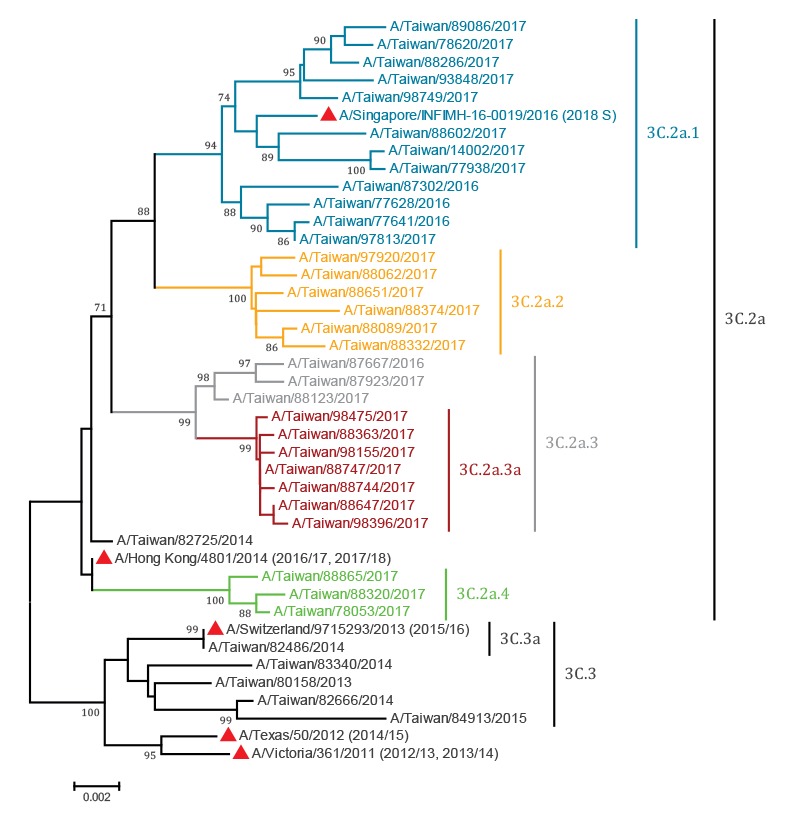
Phylogenetic relationship and amino acid changes of the influenza A(H3N2) virus haemagglutinin gene, Taiwan, 1 July 2016–15 July 2017

Analysis of haemagglutinin (HA) sequences indicated co-circulation of multiple variants in clade 3C.2a (Figure 1): 3C.2a.1 (n = 317; characterised by N171K substitution with some carrying K92R or N121K), 3C.2a.2 (n = 163; characterised by T131K and R142K substitutions), and 3C.2a.4 (n = 68; characterised by R142G and S144R substitutions, with D53N, I192T, Q197H occasionally found in some isolates). Variant 3C.2a.3 is characterised by N121K and S144K substitutions. We found a total of 153 isolates in this group among which we noticed a distinct group of variants also carrying T135K and R150K (n = 109) while the remainder were clade 3C.2a.3 isolates (n = 44; characterised by N121K and S144K substitutions, with or without S219Y). In the absence of an official World Health Organization nomenclature and for the ease of discussion in the current manuscript, we refer to the 3C.2a.3 isolates carrying the T135K and R150K substitution as putative clade 3C.2a.3a. Isolates belonging to clade 3C.2a but without any of the characteristic substitutions mentioned above were grouped as ‘other 3C.2a’ (n = 19).

Phylogenetic trees were constructed by using the neighbour-joining method in MEGA6 software (Tamura, Stecher, Peterson, Filipski, and Kumar 2013), and 1,000 bootstrap replications were performed to evaluate the robustness. The World Health Organization (WHO)-recommended vaccine strains, including the A/Victoria/361/2011(2012/13 and 2013/14 season), A/Texas/50/2012 (2014/15 season), A/Switzerland/9715293/2013(2015/16 season), A/Hong Kong/4801/2014(2016/17 and 2017/18 season) and A/Singapore/INFIMH-16–0019/2016 (2018, southern hemisphere season) are included as reference sequences (red triangle).

Antigenic analysis showed that most of the tested viruses (85.4%; 111/130) were antigenically related to the 2016/17 and 2017/18 vaccine strain, A/Hong Kong/4801/2014, indicating no evidence to support the emergence of antigenically drifted virus groups among these clades.

Sequences in the phylogenetic analysis were deposited in the EpiFlu database of the Global Initiative on Sharing All Influenza Data (GISAID) (Table 1); the authors gratefully acknowledge the originating and submitting laboratories who contributed sequences to GISAID (www.gisaid.org).

**Table 1:** Accession numbers in the Global Initiative on Sharing All Influenza Data (GISAID) EpiFlu database used for phylogenetic analysis in this study

Accession number	Virus	Originating laboratory	Submitting laboratory
EPI556816	A/Texas/50/2012	Texas Department of State Health Services-Laboratory Services	Centers for Disease Control and Prevention, US
EPI377444	A/Victoria/361/2011	WHO Collaborating Centre for Reference and Research on Influenza, Australia	National Institute for Medical Research, UK
EPI486944	A/Taiwan/80158/2013	Centers for Disease Control, Taiwan	Centers for Disease Control, Taiwan
EPI543763	A/Switzerland/9715293/2013	National Institute for Medical Research, UK	National Institute of Infectious Diseases, Japan
EPI1077171	A/Taiwan/82486/2014	Centers for Disease Control, Taiwan	Centers for Disease Control, Taiwan
EPI539576	A/Hong Kong/4801/2014	Government Virus Unit, Hong Kong	National Institute for Medical Research, UK
EPI1077172	A/Taiwan/82666/2014	Centers for Disease Control, Taiwan	Centers for Disease Control, Taiwan
EPI1077173	A/Taiwan/82725/2014	Centers for Disease Control, Taiwan	Centers for Disease Control, Taiwan
EPI1077174	A/Taiwan/83340/2014	Centers for Disease Control, Taiwan	Centers for Disease Control, Taiwan
EPI884197	A/Taiwan/84913/2015	Centers for Disease Control, Taiwan	Centers for Disease Control, Taiwan
EPI1106226	A/Singapore/INFIMH-16–0019/2016	WHO Collaborating Centre for Reference and Research on Influenza, Australia	Centers for Disease Control and Prevention, US
EPI1077177	A/Taiwan/77628/2016	Centers for Disease Control, Taiwan	Centers for Disease Control, Taiwan
EPI1077180	A/Taiwan/93848/2017	Centers for Disease Control, Taiwan	Centers for Disease Control, Taiwan
EPI1077202	A/Taiwan/88123/2017	Centers for Disease Control, Taiwan	Centers for Disease Control, Taiwan
EPI1077187	A/Taiwan/77938/2017	Centers for Disease Control, Taiwan	Centers for Disease Control, Taiwan
EPI1077213	A/Taiwan/78053/2017	Centers for Disease Control, Taiwan	Centers for Disease Control, Taiwan
EPI1077190	A/Taiwan/88602/2017	Centers for Disease Control, Taiwan	Centers for Disease Control, Taiwan
EPI1077196	A/Taiwan/88651/2017	Centers for Disease Control, Taiwan	Centers for Disease Control, Taiwan
EPI1077185	A/Taiwan/78620/2017	Centers for Disease Control, Taiwan	Centers for Disease Control, Taiwan
EPI1077211	A/Taiwan/88744/2017	Centers for Disease Control, Taiwan	Centers for Disease Control, Taiwan
EPI1077210	A/Taiwan/88747/2017	Centers for Disease Control, Taiwan	Centers for Disease Control, Taiwan
EPI884196	A/Taiwan/87302/2016	Centers for Disease Control, Taiwan	Centers for Disease Control, Taiwan
EPI884195	A/Taiwan/77641/2016	Centers for Disease Control, Taiwan	Centers for Disease Control, Taiwan
EPI884194	A/Taiwan/87667/2016	Centers for Disease Control, Taiwan	Centers for Disease Control, Taiwan
EPI955993	A/Taiwan/87923/2017	Centers for Disease Control, Taiwan	Centers for Disease Control, Taiwan
EPI955994	A/Taiwan/97813/2017	Centers for Disease Control, Taiwan	Centers for Disease Control, Taiwan
EPI973361	A/Taiwan/88062/2017	Centers for Disease Control, Taiwan	Centers for Disease Control, Taiwan
EPI955995	A/Taiwan/88089/2017	Centers for Disease Control, Taiwan	Centers for Disease Control, Taiwan
EPI973360	A/Taiwan/97920/2017	Centers for Disease Control, Taiwan	Centers for Disease Control, Taiwan
EPI973362	A/Taiwan/14002/2017	Centers for Disease Control, Taiwan	Centers for Disease Control, Taiwan
EPI1004794	A/Taiwan/88286/2017	Centers for Disease Control, Taiwan	Centers for Disease Control, Taiwan
EPI1004795	A/Taiwan/88320/2017	Centers for Disease Control, Taiwan	Centers for Disease Control, Taiwan
EPI1004796	A/Taiwan/88332/2017	Centers for Disease Control, Taiwan	Centers for Disease Control, Taiwan
EPI1018313	A/Taiwan/98155/2017	Centers for Disease Control, Taiwan	Centers for Disease Control, Taiwan
EPI1018311	A/Taiwan/88363/2017	Centers for Disease Control, Taiwan	Centers for Disease Control, Taiwan
EPI1018312	A/Taiwan/88374/2017	Centers for Disease Control, Taiwan	Centers for Disease Control, Taiwan
EPI1034309	A/Taiwan/98396/2017	Centers for Disease Control, Taiwan	Centers for Disease Control, Taiwan
EPI1034308	A/Taiwan/98475/2017	Centers for Disease Control, Taiwan	Centers for Disease Control, Taiwan
EPI1034307	A/Taiwan/88647/2017	Centers for Disease Control, Taiwan	Centers for Disease Control, Taiwan
EPI1046865	A/Taiwan/88865/2017	Centers for Disease Control, Taiwan	Centers for Disease Control, Taiwan
EPI1046867	A/Taiwan/98749/2017	Centers for Disease Control, Taiwan	Centers for Disease Control, Taiwan
EPI1046866	A/Taiwan/89086/2017	Centers for Disease Control, Taiwan	Centers for Disease Control, Taiwan

UK: United Kingdom; US: United States; WHO: World Health Organization.

**Figure 2:**
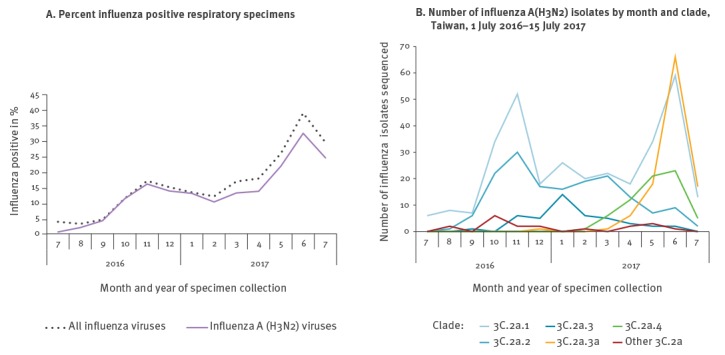
Influenza A(H3N2) viruses findings from the National Influenza Laboratory Surveillance Network, Taiwan, 1 July 2016–15 July 2017

**Figure 3:**
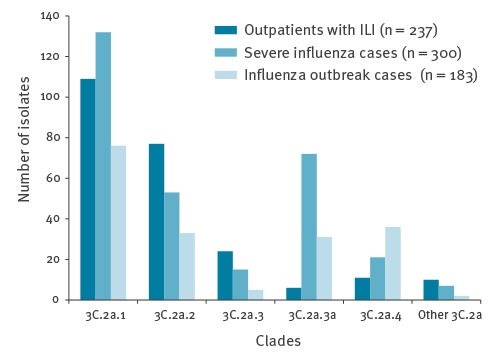
Number of influenza A(H3N2) virus isolates by clade and patient source, Taiwan, 1 July 2016–15 July 2017

Figure 2 shows the temporal distribution of clade 3C.2a virus isolates in Taiwan from 1 July 2016 to 15 July 2017. The emergence of the putative clade 3C.2a.3a and of clade 3C.2a.4 paralleled the increase of influenza positivity among respiratory specimens, indicating the start of the summer influenza epidemic. Compared with other clades, more putative 3C.2a.3a were isolated from patients with severe illness (66.1%; 72/109, p < 0.05) and more 3C.2a.4 were isolated from patients involved in outbreaks (52.9%; 36/68; p < 0.05) (Figure 3).

ILI: Influenza-like illness

## Characteristics of severe cases

We reviewed reports of 300 patients with severe influenza A(H3N2) infected with clade 3C.2a viruses. We collected data on demographics, pre-existing high-risk medical conditions for influenza (including metabolic, cardiovascular, hepatic, renal and pulmonary disorders, neuromuscular diseases, immunodeficiency, obesity with body mass index ≥ 30 kg/m^2^ and pregnancy), 2016/17 influenza vaccination status, antiviral treatment and outcome, from the National Notifiable Diseases Surveillance System in Taiwan [9]. Among the 300 patients (median age 77 years; range 0–97; IQR 62–84), 173 (57.7%) were male and 283 (94.3%) had pneumonia. Case fatality rates (n = 48; 16% overall), presence of any high-risk medical conditions mentioned above (n = 269; 89.7% overall), receipt of the 2016/17 seasonal influenza vaccine ≥ 14 days before illness onset (n = 60; 20.0% overall), and receipt of antiviral treatment within 48 hours after onset (n = 192; 64.0% overall) did not differ between patients infected with different clades (Table 2).

**Table 2:** Characteristics of patients-with severe influenza A(H3N2) by clade, Taiwan, 1 July 2016–15 July 2017^a^ (n = 300)

	3C.2a.1 (n = 132)	3C.2a.2 (n = 53)	3C.2a.3 (n = 15)	3C.2a.3a (putative) (n = 72)	3C.2a.4 (n = 21)	Other 3C.2a (n = 7)	p value^b^
Age in years	75.5 (1–97)	74.0 (50–94)	80.0 (60–90)	78.5 (1–94)	77.0 (0–91)	79.0 (18–87)	0.35
Death	23	5	2	12	4	2	0.56
High-risk medical condition^c^	121	49	14	62	16	7	0.21
Diabetes mellitus	42	20	8	19	5	1	0.24
Cardiovascular disease	50	25	8	27	8	3	0.74
Renal disease	30	12	2	13	1	0	0.27
Pulmonary disease	21	9	1	17	0	3	0.049
Vaccinated 2016/17^d^	25	11	4	14	4	2	0.97
Time from vaccination to illness onset (days)	182 (28–271)	163 (27–272)	60.5 (26–85)	231.5 (91–266)	213.5 (187–238)	100.5 (19–182)	0.02
Time from illness onset to antiviral treatment (days)	2 (0–22)	2 (1–17)	1 (0–14)	2 (0–14)	2 (0–10)	3(1–6)	0.94
≤ 48 hours after illness onset	87	33	11	46	12	3	0.75

^a^ Values are median (range) or number of patients.

^b^ Analysis of variance (ANOVA) test was used to compare medians and chi-squared test was used to compare proportions.

^c^ Conditions associated with higher risk for serious medical complications from influenza, except for hypertension. Besides, 13 of 300 patients had cancer, six had body mass index ≥ 30 kg/m^2^ and 2 had other immunodeficiency condition.

^d^ Receipt ≥ 14 days before illness onset.

Median time elapsed from 2016/17 seasonal influenza vaccination to illness onset was 203 days (range 19–271, IQR 75.3–229.5), and was significantly shorter in patients infected with 3C.2a.3 (60.5 days) and other 3C.2a clades (100.5 days).

None of the tested influenza A(H3N2) virus isolates showed mutations associated with neuraminidase inhibitor resistance.

## Discussion

We described the emergence of variant influenza A(H3N2) viruses in Taiwan during a summer wave of the 2016/17 influenza season. We found that multiple pre-existing (3C.2a.1, 3C.2a.2, 3C.2a.3) and emerging (putative 3C.2a.3a and 3C.2a.4) variants co-circulated, contributing to the summer influenza epidemic. Similar to what we have found in our clade 3C.2a.3 viruses, influenza A(H3N2) variants carrying N121K and S144K have been reported in the Denmark, Finland, Israel, Sweden and the UK, in 2016/17 [2-4,10]. In the report from the UK, clade 3C.2a.3 was further grouped into cluster I (carrying I58V and S219Y) and cluster II (N122D and S262N) [4]. In Taiwan, we found a group of variants within 3C.2a.3 carrying T135K and R150K; this variant (putative clade 3C.2a.3a) was more frequently isolated from patients with severe illness.

The 3C.2a variants were reported possibly related to lower 2016/17 influenza VE against A(H3N2), but clinical details and outcomes of patients remain unknown [1,2,11]. We found that characteristics and case fatality rates of patients infected with different clades were similar. Among a group of severely ill patients, mostly with pre-existing medical conditions, the clade of viruses they were infected with did not seem to change their outcome. However, although the proportion of severely ill cases receiving 2016/17 influenza vaccination was comparable, the time elapsed between vaccination and illness onset was significantly shorter among patients infected with clades 3C.2a.3 and other 3C.2a variants. This finding could be explained by the possible lower VE and shorter duration of protection against these two clades compared with other circulating ones. Nevertheless, all the described variants were susceptible to neuraminidase inhibitors, including oseltamivir and zanamivir, and current guidance continues to recommend antiviral treatment as early as possible for any patient with confirmed or suspected influenza who has severe illness or is at higher risk for complications [12].

A major concern related to the observed amino acid substitutions is whether they could cause major antigenic change and increase the potential of a large epidemic. Substitutions responsible for major antigenic change are located exclusively in HA antigenic sites A and B [13]. The substitutions we found in the putative clades 3C.2a.3a (T135K, R150K) and 3C.2a.4 (R142G, S144R) were all in antigenic site A. Position 135 has been identified as a site of accessory substitutions. Although no additional antigenic distance is acquired by addition of an accessory substitution, it corrects for directionality towards the subsequent antigenic phenotype in the antigenic maps [13]. Position 144 is associated with glycosylation of influenza A(H3N2) viruses and is adjacent to the receptor binding site, where most of the major cluster-transition substitutions are located. Because any amino acid changes in the 140–146 region of HA have been shown to be characteristic for antigenically distinct viruses of epidemic significance [3], the 3C.2a.4 variant carrying R142G and S144R substitutions may have the potential for antigenic change that warrants further monitoring in the following seasons. We found more 3C.2a.4 isolates from patients involved in outbreaks; whether these antigenic substitutions are related to virus transmission also needs further investigation.

The findings in this report are subject to at least three limitations. First, the isolates from patients in outbreaks and sentinel outpatients were collected by convenient, instead of systematic sampling, and only 32.3% (720/2,228) of all virus isolates were sequenced. Therefore, the results might not represent the circulating strains among the population as a whole. Second, clinical information was only available for patients with severe influenza therefore the results may not be applied to less severely ill patients. Finally, we did not collect data on ‘test-negative’ patients to calculate VE against influenza A(H3N2), and the impact of annual influenza vaccination campaign was not evaluated.

The emergence of influenza A(H3N2) virus variants in clade 3C.2a in Taiwan caused the summer influenza epidemic in 2017. Continued surveillance is warranted to monitor the evolution of circulating clade 3C.2a viruses and the impact on transmission and vaccine protection.
